# National Practice Patterns of VA ECMO and Left Ventricular Mechanical Unloading for Cardiogenic Shock

**DOI:** 10.1111/aor.70188

**Published:** 2026-07-09

**Authors:** Dustin M. Anderson-Bell, Morgan M. Millar, Rachel R. Codden, Stavros Drakos, Craig Selzman, Chloe R. Skidmore, Margaret Carlson, Frederick Welt, Tom Hanff, Hiroshi Kagawa, Jason Glotzbach, Andrea Steely, Vikas Sharma, Matt Goodwin, Daniel Haase, Jason Carr, Joseph E. Tonna

**Affiliations:** 1 Division of Respiratory, Critical Care, and Occupational Pulmonary Medicine, Department of Medicine, University of Utah Health, Salt Lake City, Utah, USA; 2 Division of Epidemiology, Department of Medicine, University of Utah School of Medicine, Salt Lake City, Utah, USA; 3 Division of Cardiovascular Medicine, Department of Medicine, University of Utah Health, Salt Lake City, Utah, USA; 4 Nora Eccles Harrison Cardiovascular Research and Training Institute (CVRTI), University of Utah School of Medicine, Salt Lake City, Utah, USA; 5 Division of Cardiothoracic Surgery, Department of Surgery, University of Utah Health, Salt Lake City, Utah, USA; 6 Department of Emergency Medicine, University of Utah Health, Salt Lake City, Utah, USA; 7 Division of Pulmonary, Critical Care, and Occupational Pulmonary Medicine, Intermountain Medical Center, Murray, Utah, USA

**Keywords:** cardiogenic shock, critical care, extracorporeal membrane oxygenation, left ventricular mechanical unloading, shock team

## Abstract

**Background:**

Cardiogenic shock (CS) remains highly morbid despite significant advancements in management. A cornerstone of this management is Venoarterial Extracorporeal Membrane Oxygenation (VA ECMO). However, consensus surrounding the management of VA ECMO for CS, including the use of concomitant Left Ventricular Mechanical Unloading (LVMU), remains limited.

**Methods:**

We thus conducted a national survey of practice patterns in the treatment of CS to characterize such variability. Surveys were distributed to physicians involved in management of VA ECMO across a variety of center types.

**Results:**

Responses representing 67 institutions were analyzed. VA ECMO was used for a median of 10% of CS patients at each institution. Formal shock teams were present at 64.1% of centers and were associated with both higher annual VA ECMO volume (P⟨0.01) and greater intra-aortic balloon pump use prior to VA ECMO (*p* = 0.03). LVMU was employed by 63.4% of centers, most commonly using Impella (93.3%), with unloading initiated at ECMO cannulation in a median of 30% of cases. Triggers and targets for LVMU varied widely, though pulmonary capillary wedge pressure was the most common endpoint.

**Conclusions:**

These findings highlight substantial heterogeneity in CS diagnosis, VA ECMO initiation, and LVMU strategies, underscoring the need for prospective studies to define optimal care.

## 1 ∣ Background

Cardiogenic shock (CS) is a highly morbid condition resulting from depressed cardiac output and inadequate perfusion [1-4]. Despite advancements in medical care, the mortality from CS has remained largely unchanged over the last twenty years. With significant advancements in device technology including increasing types and availability of peripherally inserted ventricular assist devices (pVADs), the development of centrifugal pumps, polymethylpentene (PMP) hollow fiber oxygenators, and novel cannulae (for extracorporeal membrane oxygenation [ECMO]), mechanical circulatory support (MCS) has taken an increasingly important role in the treatment of CS, with multiple clinical trials completed [5-7] and ongoing [8-12].

These recent clinical trials have not had a consistent direction of effect with both positive and negative studies. However, while all share the use of MCS as an intervention for CS or cardiac arrest, the trials differ not just in their outcomes, but in their design—including the population, severity of illness at enrollment, adjunctive interventions and treatments, and MCS devices utilized. Notable examples include the ECMO-CS Trial, which did not demonstrate benefit with the use of Venoarterial (VA) ECMO for late-stage cardiogenic shock (Society for Cardiovascular Angiography and Interventions [SCAI] Stage D/E) [6]; meanwhile the DANGER SHOCK Trial showed a benefit to the use of pVADs in myocardial infarction (MI) related CS [13]. For patients with cardiac arrest, the use of ECMO as a resuscitative measure, known as extracorporeal cardiopulmonary resuscitation (ECPR), has demonstrated mixed results. Taken together with increasing adoption of both pVADs and ECMO for CS, there has been increasing interest in the question of not just *if* MCS should be used during the treatment of refractory CS, but *when* and *how*.

In addition to the diversity of clinical trials for CS, observational data clearly demonstrate practice variability in the treatment of CS [14-19]. In response to this variability and the known difficulty in enrolling patients in clinical trials, this study aims to characterize real-world practice patterns in the treatment of CS in order to identify areas of variability and convergence. For this survey-based study, we sought to understand recognition of CS, timing and frequency of use of VA ECMO for CS, use of a dedicated shock team, specialties involved in placement and management of VA ECMO, and the use and management of adjunctive left ventricle (LV) mechanical unloading (LVMU) including initiation timing, modality, and management targets.

## 2 ∣ Methods

### 2.1 ∣ Study Participants

The target population for our survey was physicians who perform cannulation for MCS and/or provide clinical care for patients with CS throughout the United States. We searched within publicly available directories for these departments among U.S. academic medical centers and large regional referral hospitals. Candidate medical centers were identified by searching the Association of American Medical Colleges, the American Hospital Association, the Extracorporeal Life Support Organization (ELSO), and the Cardiogenic Shock Working Group (CSWG). Two eligible participants within each medical center were chosen by first identifying the institutional ECMO program directors or members of relevant divisions (cardiology, cardiothoracic surgery) noted as being on the ECMO team. If more than two individuals were identified then those within the department of cardiothoracic surgery were selected first for consistency. Contact information for these individuals was obtained from the website of their medical center or from the Cardiothoracic Surgical Trials Network (CTSNet). All identified candidate physicians were then invited to participate. Multiple responses from a single institution were permitted in order to capture practice variation within centers with the intention to include each participant's response as a unique datum in the analyses.

### 2.2 ∣ Survey Development

The initial survey was drafted by the study investigators (J.E.T., S.D., C.S.) based on their clinical experience and on the existing literature. The survey instrument underwent iterative revision with input from the Survey Design and Measurement Core at the University of Utah to improve content clarity and face validity prior to dissemination.

### 2.3 ∣ Survey Administration and Data Collection

Data were collected using an evidence-based, mixed-mode recruitment strategy. Potential participants first received an initial email (if publicly available) notifying them that they would be invited to participate in a national survey of clinicians who manage patients with cardiogenic shock. Following this email, a subsequent mailed packet was sent to these physicians' professional mailing address, which included a cover letter serving as documentation of informed consent, a $2 cash token of appreciation, and a link to the web-based survey hosted on the REDCap platform (Research Electronic Data Capture; Vanderbilt University). Completion of the online survey implied consent to participate. Thirty days after initial packets were mailed, follow-up emails inviting participation were sent to non-respondents. This email reminder process was repeated. If there was still no response by nine months from at least one participant from a given institution in the first pool of invited participants, then another candidate participant was identified from the institutional website to constitute a second pool of respondents in an attempt to increase sample size. This second search allowed for expanded eligibility, which then included physicians from pulmonary, cardiology, critical care, anesthesiology, and emergency medicine even if they were not listed as having explicit specialization in ECMO. If no response was achieved from the initial mailing to the second pool of candidate participants by one month after attempted contact, follow-up emails were sent.

### 2.4 ∣ Statistical Analyses

Descriptive statistics were used to evaluate relationships between relevant variables. Dependent variables for analyses included (1) SCAI stage at VA ECMO initiation and (2) Percent and timing of CS cases receiving VA ECMO/LVMU/Invasive hemodynamic monitoring. Independent variables included (1) presence of shock team, (2) annual volume of VA ECMO, and (3) triggers for LVMU. Chi-squared tests were used for categorical independent predictors with dichotomous outcomes and Wilcoxon–Mann–Whitney for differences between those with continuous outcomes. Kruskal–Wallis testing was used for ordered categorical predictors with continuous outcomes. A *p*-value of < 0.05 was considered statistically significant. Response rates were calculated as total number of partially and fully completed surveys, divided by the number of eligible cases, consistent with RR2 from the American Association for Public Opinion Research (AAPOR) guidelines [20]. A sensitivity analysis was conducted utilizing only one respondent from each center to assess for impact from more heavily weighting centers with multiple respondents. Additionally, characteristics of respondents from pool 1 versus pool 2 were analyzed separately (Table S1). All statistical analyses were performed using Stata Version 18 (College Park, IL, USA).

## 3 ∣ Results

### 3.1 ∣ Response Rate and Participant Characteristics

For the purpose of identifying response rate, respondents self-identified by name and institution. Of the 140 contacted institutions with 414 contacted individual candidate participants, there were 80 total responses (57 from the first pool of eligible participants, 23 from the second pool). Two respondents submitted two separate entries. Only one entry was retained for analysis, leaving 78 unique individual responses. For one individual, there was significantly greater completion in one of their responses prompting its inclusion. For the other individual, both entries had equal levels of completion and so the newer response was included. Two entries did not include the name of the respondent nor institution. Overall, with the exclusion of multiple responses from any given participant and, assuming that the entries with missing names of respondent and medical centers came from unique individuals and centers, this yielded a response rate of 18.8% (78/414) for individuals and 47.9% (67/140) of institutions. All major geographic regions were represented (Northeast 32.9%, West 19.7%, Southeast 21.1%, Midwest 17.1%, Southwest 9.2%). There was a significant bias toward cardiothoracic surgeons as respondents (*n* = 64; 84%) with the remainder being anesthesiologists, pulmonary/emergency intensivists, general surgeons, vascular surgeons, and cardiologists (Table 1). Diversity in specialty increased mildly in the second pool of respondents (Table S1).

### 3.2 ∣ Definition of Cardiogenic Shock

Of the respondents who diagnose CS based on a single operational definition, the most common criteria (24.4%) were from the 1999 SHOCK trial (which requires systolic blood pressure of < 90 mmHg for at least 30 min or the need for supportive measures to maintain a systolic blood pressure of ≥ 90 mmHg); end-organ hypoperfusion (cool extremities or a urine output of < 30 mL per hour, and a heart rate of ≥ 60 beats per minute); cardiac index of no more than 2.2 L per minute per square meter of body-surface area; and a pulmonary capillary wedge pressure of at least 15 mmHg [16, 17, 21]. However, 44.8% of participants responded “it varies”; among this response, 62.9% used lactate elevation, 57.1% used progressive shock despite optimal medical management, and 48.6% used the need for both inotropes and vasopressors (Table 2).

### 3.3 ∣ Use of a Formal Shock Team

64.1% of centers report using a formal shock team. Among those with a shock team, we found a significantly greater frequency of CS cases treated with VA ECMO per year (*p* < 0.01) as well as the use of IABP before initiation of VA ECMO (*p* = 0.03) compared with centers without a shock team. All other tested outcomes showed no significant associations with the presence of a shock team (Table 3).

### 3.4 ∣ Use of VA ECMO

Among responding institutions, 92.3% report some use of VA ECMO to support patients with acute CS. Of those who use VA ECMO, 50.0% estimate a frequency of > 12 patients placed on VA ECMO per year. The most frequent triggers for consideration of VA ECMO in the treatment of CS were clinical worsening despite optimal management (86.1%), hypoperfusion (77.8%), and need for pVAD (66.7%) (Table 2).

Respondents report that at their center, a median of 10% (interquartile range [IQR] 5, 25) of patients with CS are typically supported with VA ECMO, with 50.0% and 44.4% of institutions typically using it for SCAI Stage C and Stage D or greater, respectively. The only variable we found to be associated with center-level annual frequency of VA ECMO was Impella use prior to VA ECMO (*p* = 0.03). All other relationships examined did not reach statistical significance, including early (SCAI ≤ C) versus late (SCAI ≥ D) initiation of VA ECMO (*p* = 0.65) (Table 4).

### 3.5 ∣ Management of VA ECMO

Among responding institutions, 95.8% have an individual or team that can place VA ECMO at all times. Of these, the teams or services that can place VA ECMO include cardiothoracic surgery (97.3%), interventional cardiology (31.1%), and critical care (18.9%). Meanwhile, primary day-to-day management of VA ECMO was more evenly split between cardiothoracic surgery (47.3%) and critical care (40.5%) with a minority (9.5%) being managed by cardiology, and none by interventional cardiology. Two (2.7%) respondents answered “other” with clarification that management was either by cardiothoracic surgery or cardiology, depending on the patient, or by an ECLS consulting service.

### 3.6 ∣ Use of LV Unloading

63.4% of respondents report using LVMU for patients on VA ECMO for CS. LVMU devices are most commonly placed by cardiothoracic surgery (72.6%) and interventional cardiology (67.1%).

**FIGURE 1 ∣:**
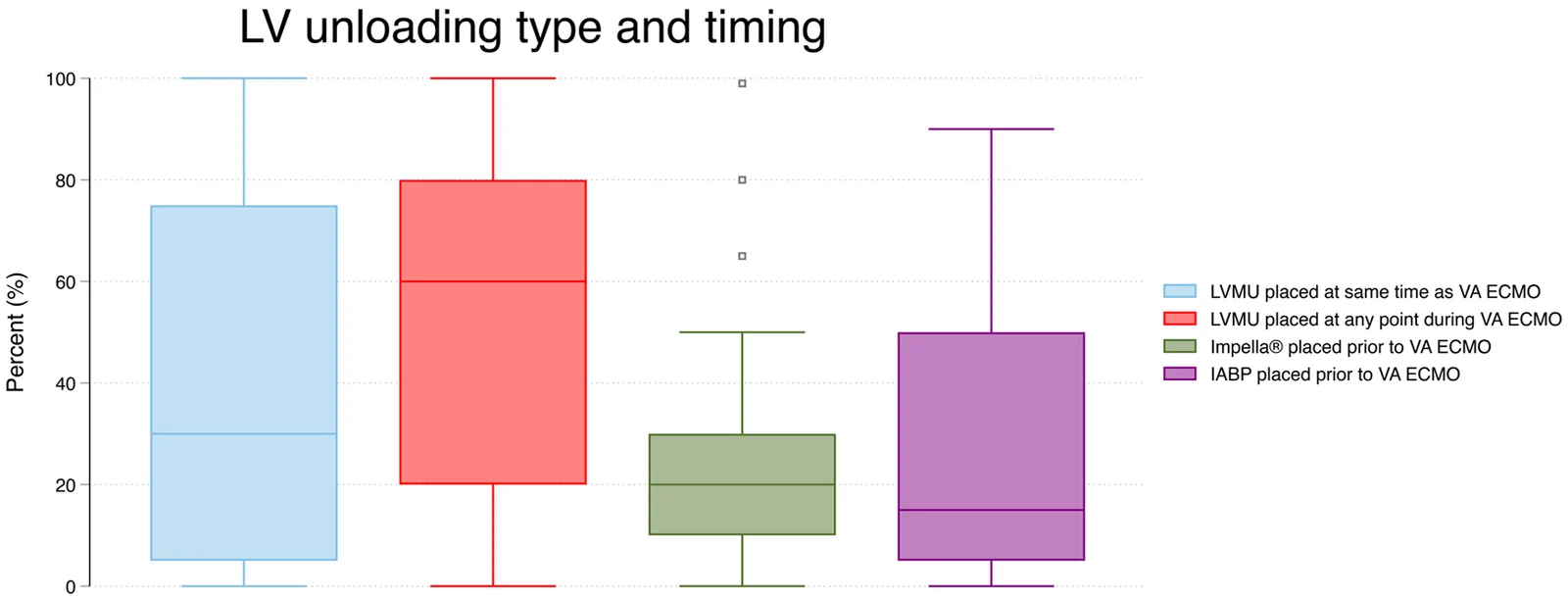
Different timing and modalities of LVMU (Percent of patients by center).

Among LVMU devices considered for use, 93.3% of centers use an Impella device and 42.2% use an intra-aortic balloon pump (IABP). Of patients receiving VA ECMO for CS, the median proportion who have LVMU placed at the time of initiation of VA ECMO across all centers is 30% (5, 75), though up to 60% (20, 80) eventually receive LVMU at some point in their VA ECMO course (Figure 1).

Common indications for LVMU include lack of aortic valve opening (51.3%), lack of arterial catheter pulsatility (48.7%; median threshold 10 mmHg [10, 16.25]), and elevated LV end diastolic diameter (LVEDd; 34.6%; median 6 cm [5.25, 6]) (Table 5).

### 3.7 ∣ LV Unloading Endpoints

**FIGURE 2 ∣:**
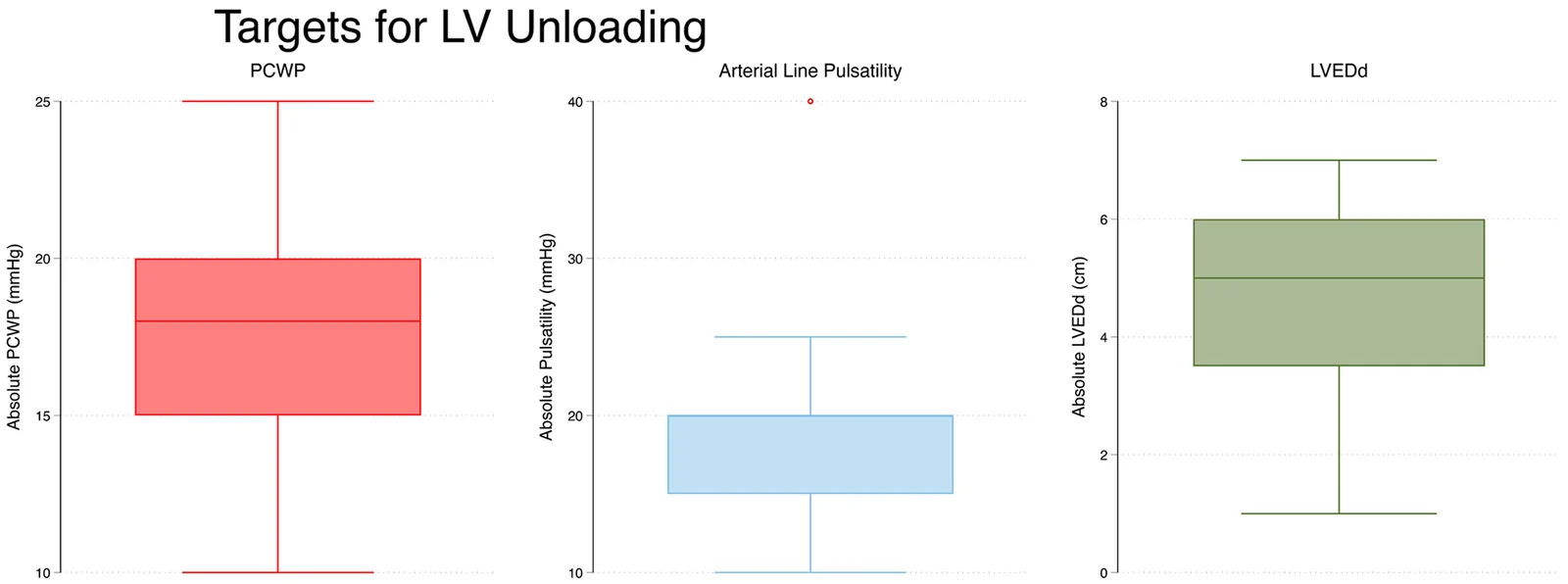
Three most common targets for LVMU (Percent of centers).

**FIGURE 3 ∣:**
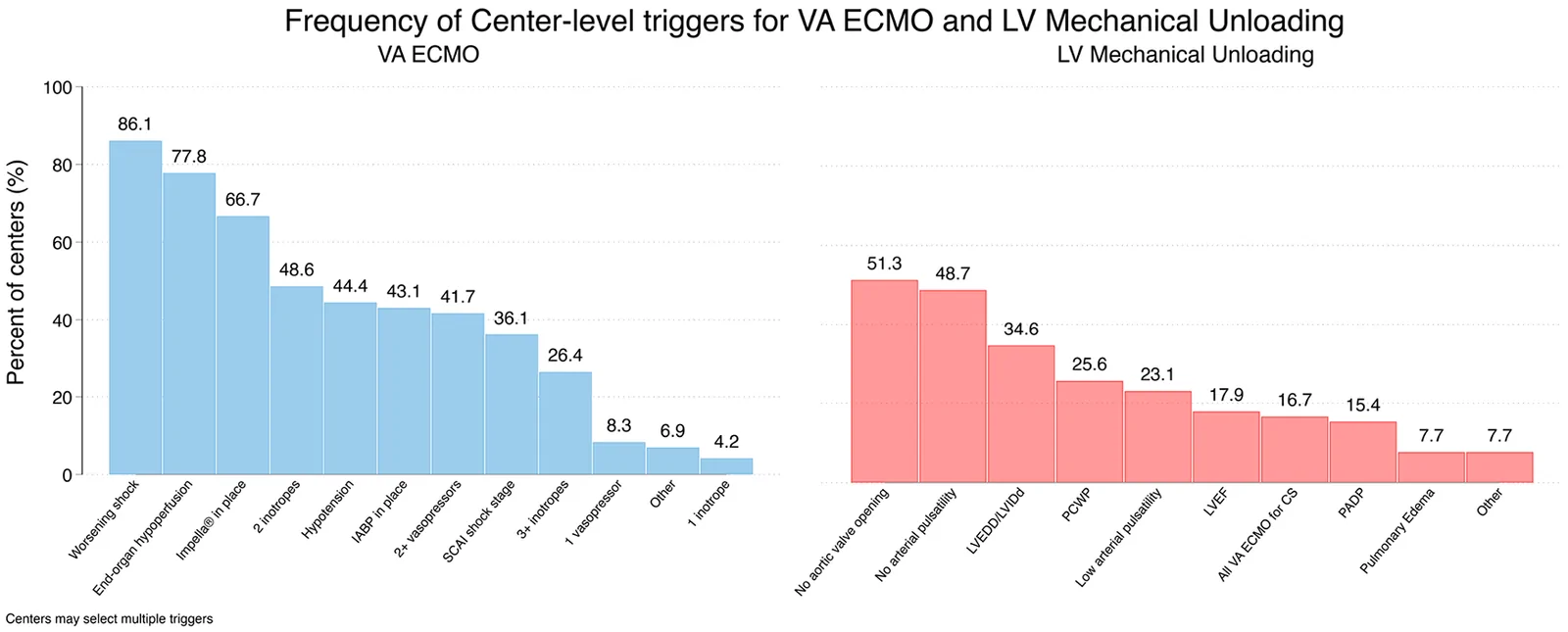
Indications for initiation of VA ECMO and LVMU (Percent of centers; selected all that apply).

A median of 95% of patients (IQR 80, 100) on VA ECMO for CS have a pulmonary artery catheter (PAC) in place during the first few days of VA ECMO. Management targets for efficacy of LVMU considered to be of greatest importance were the absolute pulmonary capillary wedge pressure (PCWP) (30.8%), absolute LVEDd/LV internal diastolic diameter (LVEDd/LVIDd) (15.4%), and absolute arterial pulsatility (14.1%) (Figure 2,3). Of those who chose a clinical/physiologic value for initiating LVMU that was also an endpoint for efficacy (i.e., absence of arterial pulsatility, PCWP < X, or LVEDd > X; *n* = 54), only 63% actually felt the physiologic value that triggered initiation was also the most important target for assessing efficacy of LVMU. Of respondents who chose a different target, 53% reported PCWP as the most important. Similarly, of those who had chosen triggers for initiating LVMU that were not available options for endpoints (i.e., left ventricular ejection fraction, pulmonary artery diastolic pressure, aortic valve opening, pulmonary edema, all VA ECMO patients; *n* = 57) 46% chose PCWP as the most important target. The mean goal for PCWP among all who chose it as the most important target was an absolute value of 17.4 ± 3.9 mmHg or a decrease by 10.6 ± 5.6 mmHg (Table 5).

### 3.8 ∣ Sensitivity Analysis

Results remained largely unchanged in the sensitivity analysis that included only one respondent per center with the exception of the association observed in the main analysis between presence of shock team and use of IABP prior to ECMO use (Tables S2-S4).

## 4 ∣ Discussion

Our survey sampled a wide distribution of large regional and academic US centers who routinely care for patients with CS, providing a likely representative depiction of the landscape of CS management. Respondent and nonrespondent hospitals were broadly distributed across the United States, although a modest geographic difference was observed in the Intermountain West, where hospitals were less likely to respond than those in other regions. Descriptively, 25.0% of hospitals in the Intermountain West responded, compared with 43.7% of hospitals in all other states. Name-based institutional patterns also suggested that respondent hospitals were somewhat more likely to be academic centers, with 48.0% of hospitals containing “University” in the name responding versus 35.5% of hospitals without “University” in the name. In contrast, hospitals containing “Medical Center” in the name were less likely to respond (34.7%) than hospitals without that designation (48.1%). These findings should be interpreted cautiously, as they are based on geographic location and hospital-name characteristics available in the file rather than more detailed structural hospital attributes.

While there are several consistent features of CS management across US hospitals such as the use of VA ECMO for select patients with CS (particularly when refractory to medical management) and invasive hemodynamic assessments peri-initiation of VA ECMO, most aspects of care were heterogeneous in our cohort.

We identified significant variability in the diagnosis of CS itself, with most centers using ill-defined criteria; these included the need for vasopressors, elevated lactate, disease progression despite optimal management, and evidence of poor perfusion on exam. It is conceivable that this dispersion may have converged more significantly around common criteria had a greater response rate been achieved. We note, however, that this variability is consistent with current guidance from the Academic Research Consortium Shock Statement (SHARC) which suggests clinical use of a broad definition of CS as “a cardiac disorder that results in both clinical and biochemical evidence of sustained tissue hypoperfusion irrespective of underlying blood pressure” [22]. The heterogeneity in this finding may thus just reflect the varied views of representative physicians in a disease state with its own inherent variability.

Our survey assessed the severity of CS at which centers initiate ECMO and our findings are largely consistent with a previous publication describing the use of SCAI stage at initiation of VA ECMO for CS. Jentzer et al. previously reported data from the international ELSO Registry showing that the distribution of SCAI Stages within 6 h prior to VA ECMO initiation was: 6.8% SCAI B, 62.1% SCAI C, 24.6% SCAI D, and 6.6% SCAI E [23]. These data suggest lower use for CS stages SCAI C and below in our survey compared to that reported by Jentzer et al. This discordance may be at least partially explained by inter-rater variability in SCAI staging in its own right [24]. Otherwise, our findings are consistent with Jentzer et al., especially considering that the previous ELSO analysis utilized the “drug+device” SCAI calculation first described by Thayer et al. [25] which, while validated, could underestimate SCAI stage given the lack of other physiologic and clinical variables in the simplified calculation.

We found that the presence of formalized shock teams was inconsistent, used by only 64.1% of institutions. Our finding that those who do use a shock team tend to have a frequency of ECMO use comports with multiple centers' reported experience that, upon implementation of a shock team, their annual rate of ECMO subsequently increased [26, 27]. A prior multicenter observational study found that centers using shock teams were more likely to obtain invasive hemodynamics, use “advanced” types of LVMU (beyond IABP), and have lower rates of intensive care unit (ICU) mortality for CS [28]. Although these relationships were not borne out in our statistical analyses, our study did not quantify relative frequencies of each LVMU strategy; it is possible that relative frequency of LVMU within each participating center may differ based on their use of a shock team. Furthermore, given our lack of patient level outcome data in this present study, we are not able to examine the association of these systems with mortality and other clinical outcomes. Once the decision has been made to initiate VA ECMO, we found widespread consistency in the teams and services that cannulate for ECMO; cardiothoracic surgery is nearly universal and many institutions also utilize interventional cardiology and critical care medicine teams. After placement, primary day-to-day management is relatively evenly distributed between cardiothoracic surgery and critical care. We note though that multidisciplinary care and shared decision making were not captured in our survey, and the interpretation of “primary management of day-to-day care” of VA ECMO may differ between respondents.

Although a majority of institutions typically employ LVMU for patients with CS on VA ECMO, the type, timing, and hemodynamic goals vary dramatically. At many centers, the LVMU device was placed prior to initiation of VA ECMO, initially intended as a first MCS step, but then was retained for LVMU once patients were cannulated for VA ECMO. A median of 20% of respondents reported initial use of Impella, and an additional 20% reported IABP prior to VA ECMO. While many patients received the MCS/LVMU prior to initiation of ECMO, nearly one third underwent placement of the LVMU device concomitantly with cannulation for VA ECMO.

Of institutions employing LVMU, a large majority (93.3%) use Impella, while less than half use an IABP; far fewer use other methods such as atrial septostomy or intracardiac catheters. Though the proportion of institutions employing some form of LVMU is consistent with international data from a EuroELSO survey of LVMU practices in VA ECMO [29] the same survey found IABP to be the most commonly employed device in contradistinction to our discovered predominance of Impella. Our findings are consistent with recent data showing increasing preference for Impella as choice of MCS within the United States [30] and may reflect differences in payer models between the US and Europe.

Just as diagnosis of CS is made based on a variety of triggers, so too is the decision to implement LVMU and the targets for determining efficacy of unloading. Our findings that the most common indications for unloading were lack of arterial catheter pulsatility, lack of aortic valve opening, and echocardiographic evidence of dilated LV are also consistent with the EuroELSO survey where arterial pulsatility and LV dilation were common triggers [29]. However, in their cohort, pulmonary edema represented the third most common trigger at 64% of their respondents while this was only chosen by 8.6% of our participants. The shared predominance of arterial pulsatility and a dilated LV as triggers suggests their potential for inclusion as consensus guidelines in the future. Specific targets for efficacy of LVMU also lacked consistency, though PCWP was the common target. For those who do target PCWP, the participants in our survey seem to use similar goals to those of international colleagues in the EuroELSO survey, with respondents in both surveys reporting a median goal of 18 mmHg [15-20]. This, along with our finding that a median of 95% of patients have a PAC in place during VA ECMO initiation, demonstrates general agreement with the recommendations by The International Society for Heart and Lung Transplantation/Heart Failure Society of America Guideline on Acute Mechanical Circulatory Support for utilization of a peri-implantation PAC and choosing a device for CS based on the ability to reach multiple invasive targets, including a PCWP < 18 [31]. This overall practice diversity in LVMU reflects the variety of triggers and management strategies recommended for consideration in the 2021 ELSO Interim Guidelines for VA ECMO in Adult Cardiac Patients [32]. Furthermore, this variability may also represent a widespread lack of institution-specific protocols, a feature that is not captured here.

Our study has important limitations as survey studies are subject to multiple potential biases. We have tried to address this by our systematic sampling and recruitment approach but acknowledge that some bias may remain. For example, given that this is a voluntary survey with a relatively low response rate, it is plausible that individuals of institutions with greater interest in ECMO and CS may have been more likely to participate and that respondents may differ from non-respondents in other important ways. This may result in over-representation of higher volume centers where practice patterns may differ than those with lower volume. However, we are reassured by research indicating that there is little correlation between response rate and representativeness or nonresponse bias [33-35]. Centers with multiple respondents are also inherently over-represented as responses from each participant were uniquely included in the analysis. However, doing so allowed for better representation of intra-center practice variability and sensitivity analysis did not demonstrate significantly different findings in practices nor outcomes aside from the loss of significance between presence of shock team and the use of IABP before ECMO. Meanwhile, given that each center is represented by a maximum of only two clinicians, within-center variability is not well characterized. This is particularly true of between-specialty variation as responses are strongly biased toward that of cardiothoracic surgeons who comprised the majority of participants. This source of variance may serve as an important residual confounder of the analyses. Additionally, we acknowledge a risk of recall bias given that data are self-reported and time-dependent biases given the 9-month duration of the recruitment period (though comparisons between the two pools did not demonstrate significant changes in specialty nor practice patterns).

## 5 ∣ Conclusions

In summary, our data strongly suggest variability in the diagnostic criteria of CS and the triggers for use of VA ECMO, and in the role, type, timing, and targets of LVMU while on VA ECMO. While the use of a formalized shock team is increasingly common, it is still inconsistent. These data suggest the opportunity for clinical trials comparing thresholds for initiation of VA ECMO, the use of LVMU, and different management practices during CS treatment.

## Supplementary Material

Supplementary Online Content

Survey

Additional supporting information can be found online in the Supporting Information section. **Table S1:** Differences in characteristics between 1st and 2nd pool of participants. **Table S2:** Sensitivity analysis of cardiogenic shock and VA ECMO practices. **Table S3:** Sensitivity analysis of LV mechanical unloading practices. **Table S4:** Sensitivity analysis of associations between shock team presence and VA ECMO/LV mechanical unloading practices. **Data S1:** Survey.

## Figures and Tables

**TABLE 1 ∣:** Center characteristics.

	*N* (%)
Annual VA ECMO runs for CS	*N* = 78
< 1	6 (7.7)
1–2	4 (5.1)
3–6	16 (20.5)
7–12	16 (20.5)
> 12	36 (46.2)
Region	*N* = 76
Northeast	25 (32.9)
Southeast	16 (21.1)
West	15 (19.7)
Midwest	13 (17.1)
Southwest	7 (9.2)
Specialty	*N* = 72
Cardiothoracic surgery	64 (88.9)
Anesthesiology	4 (5.6)
ICU (Pulm/EM)	5 (6.9)
General surgery	1 (1.4)
Vascular surgery	1 (1.4)
Cardiology	1 (1.4)

*Note:* Characteristics of all institutions represented in analyses.

**TABLE 2 ∣:** Cardiogenic shock and VA ECMO practices.

	*N* (%)
Definition of CS (One best)	*N* = 78
Criteria vary by case	35 (44.8)
Lactate elevation	22
Progressive shock despite optimal medical management	20
Need for both inotropes and vasopressors	17
SHOCK trial (1999) criteria^a^	19 (24.4)
SCAI stage C	9 (11.5)
Use of a shock team	*N* = 78
Yes	50 (64.1)
No	28 (35.9)
Indications for VA ECMO (All that apply)	*N* = 72
Clinical worsening despite optimal medical management	62 (86.1)
Hypoperfusion	56 (77.8)
Need for Impella	48 (66.7)
2 Inotropes	35 (48.6)
SCAI stage at VA ECMO initiation (One most common)	*N* = 72
B or greater	1 (1.4)
C or greater	36 (50.0)
D or greater	32 (44.4)
E or greater	3 (4.2)

a SHOCK Trial Criteria: All of the following: 1) Systolic blood pressure of 〈 90 mm Hg for at least 30 min or the need for supportive measures to maintain a systolic blood pressure of ≥ 90 mm Hg; 2) End-organ hypoperfusion (cool extremities or a urine output of 〈30 mL per hour, and a heart rate of ≥ 60 beats per minute); 3) Cardiac index of no more than 2.2 liters per minute per square meter of body-surface area; and 4) A pulmonary capillary wedge pressure of at least 15 mm Hg.

**TABLE 3 ∣:** Analyses of associations between shock team presence and VA ECMO/LV mechanical unloading practices.

Variable	No shock team	Shock team	
By percent of all centers, *N* (%)^a^	(*N* = 23)	(*N* = 46)	*p*
Early ECMO initiation (SCAI ≤ C)	13 (56.5%)	24 (52.2%)	0.73
Frequency of ECMO for CS			< 0.01*
1–2 cases/year	4 (17.4%)	0 (0.0%)	
3–6. cases/year	8 (34.8%)	8 (17.4%)	
7–12 cases/year	3 (13.0%)	12 (26.1%)	
> 12 cases/year	8 (34.8%)	26 (56.5%)	
By percent of patients within each center, median (IQR)^b^			
Percent of CS patients treated with ECMO	10 (5, 25)	10 (5, 25)	0.58
IABP use prior to ECMO	10 (1, 25)	20 (10, 50)	0.03*
pVAD use prior to ECMO	20 (10, 50)	20 (10, 25)	0.42
LVMU at ECMO initiation for CS	37.5 (20, 75)	27.5 (5, 70)	0.38
LVMU at any point during VA ECMO for CS	57.5 (18, 80)	55 (20, 80)	0.60
Presence of PAC early in ECMO	100 (90, 100)	95 (80, 100)	0.42

a Significance tested using *χ*^2^.

b Significance tested using Wilcoxon–Mann–Whitney.

* Statistically significant at *p* < 0.05.

**TABLE 4 ∣:** Analyses of annual ECMO volume and ECMO/LV mechanical unloading practices.

Measure	1–2 cases/year (*N* = 4)	3–6 cases/ year (*N* = 16)	6–12 cases/ year (*N* = 16)	> 12 cases/ year (*N* = 35)	*p*
LVMU at ECMO start	25.5 (0.5, 25.5)	37.5 (7.5, 77.5)	42.5 (12.5, 70)	25 (10, 70)	0.55
LVMU any point on ECMO	9.5 (0.5, 46.5)	50 (20, 75)	72.5 (27.5, 92.5)	50 (25, 80)	0.15
Impella before ECMO	50 (30, 74.5)	20 (10, 40)	25 (20, 50)	10 (10, 25)	0.03*
IABP before ECMO	15 (2.5, 52.5)	25 (1.5, 50)	27.5 (10, 80)	10 (5, 25)	0.40
Early ECMO (SCAI ≤ C); *n* (% of centers)	2 (50.0)	10 (62.5)	9 (56.3)	16 (44.4)	0.65

*Note:* Values are reported as median (IQR) unless otherwise stated. Significance tested using Kruskal–Wallis test.

* Statistically significant at *p* < 0.05.

**TABLE 5 ∣:** LV mechanical unloading practices.

	*N* = 71
Institution routinely uses LVMU	45 (63.4)
If LVMU is routinely used, what method (All that apply)^a^	
Impella	42 (93.3)
IABP	19 (42.2)
LAVA	8 (17.8)
Atrial septostomy	7 (15.6)
Atrial catheter	6 (13.3)
LV catheter	3 (6.7)
PA vent	1 (2.2)
Surgical LV vent	1 (2.2)
Indications for LVMU (All that apply)^b^	
Lack of aortic valve opening	40 (51.3)
Lack of arterial catheter pulsatility	38 (48.7)
LVEDd/LVIDd	27 (34.6)
Most important targets for LVMU (mean reported values)^b^	
Absolute PCWP (17.4 ± 3.9 mmHg)	24 (30.8)
Absolute LVEDd/LVIDd (4.7 ± 1.8 cm)	12 (15.4)
Absolute arterial catheter pulsatility (19.5 ± 8.2 mmHg)	11 (14.1)
Decrease in LVEDd/LVIDd (3.5 ± 3.0 cm)	9 (11.5)
Other	7 (9.0)

Abbreviations: IABP, intra-aortic balloon pump; LAVA, left atrial veno-arterial transseptal cannulation; LVEDd, left ventricular end diastolic diameter; LVIDd, left ventricular internal diastolic diameter; LVMU, left ventricular mechanical unloading; PCWP, pulmonary capillary wedge pressure.

a Denominator is the number of institutions reporting routine use of LVMU.

b Denominator is all respondents.

## Data Availability

The data that support the findings of this study are openly available in Figshare at [https://doi.org/10.6084/m9.figshare.32795178].
