# Supplementary material for: National Practice Patterns of VA ECMO and Left Ventricular Mechanical Unloading for Cardiogenic Shock
Source: Artif Organs. 2026 Jul 9;50(10):1443–52. doi: 10.1111/aor.70188 (PMC13599474; doi:10.1111/aor.70188)
Supplement: Supplementary file 1 [file AOR-50-1443-s002.pdf]

Thank you for your willingness to complete this survey! Please note that this form will NOT save automatically. For your responses to be recorded, you need to either click "Submit" or "Save and Return Later" at the bottom of the screen. The best option is to complete the survey in full in one sitting (it should only take a few minutes to complete), but if you opt to save and return later, you will be able to re-enter the link and pick up where you left off.

Name ______________________________

Institution ______________________________

This question asks how you identify cardiogenic shock at your center/clinical group. As examples, we list the criteria from five clinical trials, SCAI stages, and INTERMACS stages. Please pick the one you use, or that approximates what you use.

Which one of the following criteria does your Department/Clinical Group use for diagnosing acute cardiogenic shock?

- ○ SHOCK Trial (1999): SBP < 90 mmHg for >30 minutes or vasopressor support to maintain SBP >90 mmHg; Evidence of end-organ damage (UO < 30mL/h or cool extremities); Hemodynamic criteria: CI < 2.2 and PCWP >15 mmHg
- ○ IABP-SOAP II (2012): MAP < 70 mmHg or SBP < 100 mmHg despite adequate fluid resuscitation (at least 1L of crystalloids or 500mL of colloids); Evidence of end-organ damage (AMS, mottled skin, UO < 0.5mL/kg for 1 hour, or serum lactate >2 mmol/L)
- ○ EHS-PCI (2012): SBP < 90 mmHg for 30 minutes or inotrope use to maintain SBP >90 mmHg; Evidence of end-organ damage and increased filling pressures
- ○ ESC-HF Guidelines (2016): SBP < 90 mmHg with appropriate fluid resuscitation with clinical and laboratory evidence of end-organ damage; Clinical: cold extremities, oliguria, AMS, narrow pulse pressure; Laboratory: metabolic acidosis, elevated serum lactate, elevated serum creatinine
- ○ KAMIR-NIH (2018): SBP < 90 mmHg for >30 minutes or supportive intervention to maintain SBP >90 mmHg; Evidence of end-organ damage (AMS, urine output < 30 mL/h, or cool extremities)
- ○ SCAI Stage B
- ○ SCAI Stage C
- ○ SCAI Stage D
- ○ SCAI Stage E
- ○ INTERMACS 1
- ○ INTERMACS 2
- ○ INTERMACS 3
- ○ INTERMACS 4
- ○ INTERMACS 5
- ○ INTERMACS 6
- ○ INTERMACS 7
- ○ It varies

If "it varies," which 3 features of these criteria make you most concerned for the presence of acute cardiogenic shock in your unit? (Please choose exactly 3.)

- ☐ 1 inotrope
- ☐ ≥2 inotropes
- ☐ Both inotropes and vasopressors
- ☐ Lactate elevation, presumed due to acute cardiogenic worsening
- ☐ Acute renal insufficiency, presumed due to acute cardiogenic worsening
- ☐ Hypoperfusion on physical exam, presumed due to acute cardiogenic worsening
- ☐ Hypotension, presumed due to acute cardiogenic worsening
- ☐ Use of IABP
- ☐ Use of Impella
- ☐ Progressive cardiogenic shock, despite optimal medical management
- ☐ Cardiac arrest
- ☐ Other (please specify)
- ☐ Not sure

Other, please specify.

____

Does your institution/clinical group use a shock team approach for cardiogenic shock?

- ◯ No
- ◯ Yes
- ◯ Other

Other, please specify.

____

Does your institution/clinical group ever use VA ECMO to support patients with acute cardiogenic shock (not post-operative)?

- ◯ No
- ◯ Yes

Thank you for participating! Please click the "submit" button below.

About how many times per year are patients placed on VA ECMO for acute cardiogenic shock (CS) at your center?

- ◯ < 1 time
- ◯ 1-2 times
- ◯ 3-6 times
- ◯ 7-12 times
- ◯ >12 times

Approximately what % of patients with acute cardiogenic shock are supported with VA ECMO at your center?

____
(%)

Which clinical conditions/situations/devices would trigger consideration of initiation of VA ECMO for acute cardiogenic shock at your institution/clinical group? (Select all that apply)

- ☐ IABP
- ☐ Impella
- ☐ Clinical worsening despite optimal medical management
- ☐ 1 inotrope
- ☐ 2 inotropes
- ☐ 3 inotropes
- ☐ 1 vasopressor
- ☐ 2 vasopressors
- ☐ Hypoperfusion
- ☐ Hypotension
- ☐ SCAI stage
- ☐ Other (please specify)

Other, please specify.

____

At what SCAI stage does your institution/clinical group typically place ECMO? (Please refer to the description of stages below.)	◯ SCAI Stage B or greater<br>◯ SCAI Stage C or greater<br>◯ SCAI Stage D or greater<br>◯ SCAI Stage E

SCAI SHOCK STAGE	PHYSICAL EXAM	BIOCHEMICAL MARKERS	HEMODYNAMICS
A	Normal JVP<br>Lung souds clear<br>Strong distal pulses<br>Normal mentation	Normal renal function<br>Normal lactic acid	Normotensive (SBP ≥ 100 or normal for pt).<br>If hemodynamics done:<br>Cardiac index ≥ 2.5<br>CVP < 10<br>PA Sat ≥ 65%
B	Elevated JVP<br>Rales in lung fields<br>Strong distal pulses<br>Normal mentation	Normal lactate<br>Minimal renal function impairment<br>Elevated BNP	SBP < 90 OR MAP < 60 OR > 30 mmHg drop<br>Pulse ≥ 100<br>If hemodynamics done:<br>Cardiac index ≥ 2.2<br>PA Sat ≥ 65%
C	Ashen, mottled, dusky<br>Volume overload<br>Extensive Rlaes<br>Killip class 3 or 4<br>BiPap or mechanical ventilation<br>Acute alteration in mental status	Lactate ≥ 2<br>Creatinine doubling OR >50% drop in GFR<br>Increased LFTs<br>Elevated BNP<br>Urine Output <30ml/h	Drugs/device used to maintain BP above stage B values.<br>Cardiac index < 2.2<br>PCWP > 15<br>RAP/PCWP ≥ 0.8<br>PAPI < 1.85<br>Cardiac Power Output ≤ 0.6
D	Any of Stage C	Any of stage C<br>AND<br>deteriorating	Any of stage C<br>AND<br>Requiring multiple pressors OR addition of mechanical circulatory support devices to maintain perfusion
E	Near pulselessness<br>Cardiac collapse<br>Mechanical ventilation<br>Defibrillator used	Lactate ≥ 5<br>pH ≤ 7.2	No SBP without resuscitation<br>PEA or Refractory VT/VF<br>Hypotension despite maximal support

Baran DA, Grines CL, Bailey S, et al. SCAI clinical expert consensus statement on the classification of cardiogenic shock. Catheterization and Cardiovascular Interventions, May 19, 2019. https://doi.org/10.1002/ccd.28329

Are there individuals or a team that can place VA ECMO 24/7 within your institution/clinical group?	◯ No<br>◯ Yes
Which specialties place VA ECMO for cardiogenic shock at your center per hospital policy/clinical practice?	☐ Cardiology<br>☐ Interventional cardiology<br>☐ Critical care<br>☐ Cardiothoracic surgery<br>☐ General surgery<br>☐ Vascular surgery<br>☐ Other (please specify)
Other, please specify.	__________________________________
Which specialty primarily manages the day to day care of VA ECMO for CS at your center?	◯ Cardiology<br>◯ Interventional cardiology<br>◯ Critical care<br>◯ Cardiothoracic surgery<br>◯ General surgery<br>◯ Vascular surgery<br>◯ Other
Other, please specify.	__________________________________
What % of patients at your institution who receive VA ECMO for cardiogenic shock have an IABP first?	__________________________________<br>(%)
What % of patients at your institution who receive VA ECMO for cardiogenic shock have an Impella first?	__________________________________<br>(%)
What % of patients at your institution who receive VA ECMO for cardiogenic shock have mechanical LV unloading placed at the same time ECMO is placed?	__________________________________<br>(%)
What % of patients at your institution who receive VA ECMO for cardiogenic shock have mechanical LV unloading placed at any point while they are supported on ECMO (including right away)?	__________________________________<br>(%)
Does your institution typically use LV decompression for patients on VA ECMO for medical cardiogenic shock?	◯ No<br>◯ Yes
If yes, what approach is used? (Select all that apply.)	☐ Impella<br>☐ IABP<br>☐ Atrial septostomy/puncture<br>☐ Direct catheter into left atrium (LA)<br>☐ Direct catheter into left ventricle (LV)<br>☐ Transseptal left atrial cannulation (LAVA) ECMO<br>☐ Other (please specify)
Other, please specify.	__________________________________

What indications warrant decompression at your institution/clinical group? (Select all that apply.)	☐ Lack of pulsatility on arterial line<br>☐ Low pulsatility on arterial line<br>☐ All patients with VA ECMO for cardiogenic shock<br>☐ LV end diastolic diameter/LVIDd size<br>☐ Ejection fraction<br>☐ PCWP<br>☐ Pulmonary diastolic pressure<br>☐ Lack of aortic valve opening<br>☐ Other (please specify)
If low pulsatility on arterial line, less than what SBP-DBP difference in mmHg?	______________________________<br>(mmHg)
If LV end diastolic diameter/LVIDd size, greater than what cm?	______________________________<br>(cm)
If ejection fraction, less than what percentage?	______________________________<br>(%)
If PCWP, greater than what mmHg?	______________________________<br>(mmHg)
If pulmonary diastolic pressure, greater than what mmHg?	______________________________<br>(mmHg)
Other, please specify.	______________________________
Which specialty at your institution/clinical group performs decompression according to policy/practice? (Select all that apply.)	☐ Cardiology<br>☐ Interventional cardiology<br>☐ Critical care<br>☐ Cardiothoracic surgery<br>☐ General surgery<br>☐ Vascular surgery<br>☐ Other (please specify)
Other, please specify.	______________________________

How do you as an individual define effective LV decompression on VA ECMO? Rank 1-3, 1 being most important, 3 being least important.

1: Most important	○ PCWP below ______ mmHg<br>○ Decrease in PCWP of at least ______mmHg<br>○ LV end diastolic diameter (LVIDd) of < ______ cm<br>○ Decrease in LV end diastolic diameter (LVIDd) of at least _____ cm<br>○ Arterial pulsatility (SBP-DBP) of at least _____ mmHg<br>○ Increase in arterial pulsatility (SBP-DBP) of at least _____ mmHg<br>○ Other (please specify)

PCWP below ______ mmHg

____
(mmHg)

Decrease in PCWP of at least _____ mmHg

____
(mmHg)

LV end diastolic diameter (LVIDd) of < _____ cm

____
(cm)

Decrease in LV end diastolic diameter (LVIDd) of at least _____ cm

____
(cm)

Arterial pulsatility (SBP-DBP) of at least _____ mmHg

____
(mmHg)

Increase in arterial pulsatility (SBP-DBP) of at least _____ mmHg

____
(mmHg)

Other, please specify

____

2: Medium importance

- ◯ PCWP below ______ mmHg
- ◯ Decrease in PCWP of at least ______mmHg
- ◯ LV end diastolic diameter (LVIDd) of < ______ cm
- ◯ Decrease in LV end diastolic diameter (LVIDd) of at least _____ cm
- ◯ Arterial pulsatility (SBP-DBP) of at least _____ mmHg
- ◯ Increase in arterial pulsatility (SBP-DBP) of at least _____ mmHg
- ◯ Other (please specify)

PCWP below ______ mmHg

____
(mmHg)

Decrease in PCWP of at least _____ mmHg

____
(mmHg)

LV end diastolic diameter (LVIDd) of < _____ cm

____
(cm)

Decrease in LV end diastolic diameter (LVIDd) of at least _____ cm

____
(cm)

Arterial pulsatility (SBP-DBP) of at least _____ mmHg

____
(mmHg)

Increase in arterial pulsatility (SBP-DBP) of at least _____ mmHg

__________________ (mmHg)

Other, please specify

____

3: Least important

- ◯ PCWP below ______ mmHg
- ◯ Decrease in PCWP of at least ______mmHg
- ◯ LV end diastolic diameter (LVIDd) of < ______ cm
- ◯ Decrease in LV end diastolic diameter (LVIDd) of at least _____ cm
- ◯ Arterial pulsatility (SBP-DBP) of at least _____ mmHg
- ◯ Increase in arterial pulsatility (SBP-DBP) of at least _____ mmHg
- ◯ Other (please specify)

PCWP below ______ mmHg

__________________ (mmHg)

Decrease in PCWP of at least _____ mmHg

__________________ (mmHg)

LV end diastolic diameter (LVIDd) of < _____ cm

__________________ (cm)

Decrease in LV end diastolic diameter (LVIDd) of at least _____ cm

__________________ (cm)

Arterial pulsatility (SBP-DBP) of at least _____ mmHg

__________________ (mmHg)

Increase in arterial pulsatility (SBP-DBP) of at least _____ mmHg

__________________ (mmHg)

Other, please specify

____

What % of patients with cardiogenic shock on ECMO have a pulmonary arterial catheter during the first few days of ECMO?

__________________ (%)
