# Supplementary material for: National Practice Patterns of VA ECMO and Left Ventricular Mechanical Unloading for Cardiogenic Shock
Source: Artif Organs. 2026 Jul 9;50(10):1443–52. doi: 10.1111/aor.70188 (PMC13599474; doi:10.1111/aor.70188)
Supplement: Supplementary file 2 [file AOR-50-1443-s001.docx]

**Supplement**

**Table 1: Differences in characteristics between 1^st^ and 2^nd^ pool of participants**

|  | **Pool 1** | **Pool 2** |
| --- | --- | --- |
| **Frequency of ECMO for CS** |  |  |
| 1–2 cases/year | 4 (7.8) | 0 |
| 3–6. cases/year | 13 (25.5) | 3 (14.3) |
| 7-12 cases/year | 12 (23.5) | 4 (19) |
| >12 cases/year | 22 (43.1) | 14 (66.7) |
| **Early ECMO Initiation** | 28 (50) | 9 (42.8) |
| **Shock Team Present** | 34 (60.7) | 13 (59.1) |
| **Institution routinely uses LVMU** | 32 (57.1) | 13 (59.1) |
| **Specialty of Respondent** | **N=54** | **N=22** |
| Cardiothoracic Surgery | 49 (90.7) | 15 (68.2) |
| Anesthesiology | 1 (1.9) | 3 (13.6) |
| ICU (Pulm/EM) | 2 (3.7) | 3 (13.6) |
| General Surgery | 0 | 1 (4.6) |
| Vascular Surgery | 1 (1.9) | 0 |
| Cardiology | 1 (1.9) | 0 |

| **Table 2: Sensitivity Analysis of Cardiogenic Shock and VA ECMO Practices** | |  |
| --- | --- | --- |
|  | **Main**  **N (%)** | **Sensitivity** |
| **Definition of CS** (One best) | **N = 78** | **N = 65** |
| Criteria vary by case | 35 (44.8) | 29 (44.6) |
| Lactate elevation | 22 | 19 |
| Progressive shock despite medical management | 20 | 14 |
| Need for both inotropes and vasopressors | 17 | 14 |
| SHOCK trial (1999) Criteria* | 19 (24.4) | 16 (24.6) |
| SCAI Stage C | 9 (11.5) | 6 (9.2) |
| **Use of a Shock Team** | **N = 78** | **N = 65** |
| Yes | 50 (64.1) | 40 (61.5) |
| No | 28 (35.9) | 22 (33.9) |
| **Indications for VA ECMO** (All that apply) | **N = 72** | **N = 65** |
| Clinical worsening despite medical management | 62 (86.1) | 50 (76.9) |
| Hypoperfusion | 56 (77.8) | 45 (69.2) |
| Need for Impella® | 48 (66.7) | 39 (60.0) |
| 2 Inotropes | 35 (48.6) | 30 (46.2) |
| **SCAI Stage at VA ECMO initiation** (One most common) | **N = 72** | **N = 59** |
| B or greater | 1 (1.4) | 0 |
| C or greater | 36 (50.0) | 30 (50.9) |
| D or greater | 32 (44.4) | 26 (44.1) |
| E or greater | 3 (4.2) | 3 (5.1) |

| **Table 3: Sensitivity Analysis of LV Mechanical Unloading Practices** | |  |
| --- | --- | --- |
| Institution routinely uses LVMU | **Main**  **N = 71**  45 (63.4) | **Sensitivity**  **N = 58**  36 (62) |
| **If LVMU is routinely used, what method** (All that apply)* |  |  |
| Impella® | 42 (93.3) | 33 (91.7) |
| IABP | 19 (42.2) | 14 (38.9) |
| LAVA | 8 (17.8) | 6 (16.7) |
| Atrial Septostomy | 7 (15.6) | 5 (13.9) |
| Atrial Catheter | 6 (13.3) | 3 (8.3) |
| LV Catheter | 3 (6.7) | 3 (8.3) |
| PA Vent | 1 (2.2) | 1 (2.8) |
| Surgical LV Vent | 1 (2.2) | 1 (2.8) |
| **Indications for LVMU** (All that apply)^†^ |  |  |
| Lack of aortic valve opening | 40 (51.3) | 33 (50.8) |
| Lack of arterial catheter pulsatility | 38 (48.7) | 33 (50.8) |
| LVEDd/LVIDd | 27 (34.6) | 22 (33.8) |
| **Most Important Targets for LVMU** (mean reported values) ^†^ |  |  |
| Absolute PCWP (17.4 ± 3.9 mmHg) | 24 (30.8) | 19 (29.2) |
| Absolute LVEDd/LVIDd (4.7 ± 1.8 cm) | 12 (15.4) | 11 (16.9) |
| Absolute arterial catheter pulsatility (19.5 ± 8.2 mmHg) | 11 (14.1) | 11 (16.9) |
| Decrease in LVEDd/LVIDd (3.5 ± 3.0 cm) | 9 (11.5) | 5 (7.7) |
| Other | 7 (9.0) | 4 (6.2) |

*IABP: Intra-Aortic Balloon Pump; LAVA: Left Atrial Veno-Arterial Transseptal Cannulation; LVEDd: Left Ventricular End Diastolic Diameter; LVIDd: Left Ventricular Internal Diastolic diameter; LVMU: Left Ventricular Mechanical Unloading; PCWP: Pulmonary Capillary Wedge Pressure*

** Denominator is number of institutions reporting routine use of LVMU*

*† Denominator is all respondents*

**Table 4. Sensitivity Analysis of Associations Between Shock Team Presence and VA ECMO / LV Mechanical Unloading Practices**

| **Variable**  **By percent of all centers, N (%)**^†^ | **No Shock Team**  **(N = 23)** | **Shock Team**  **(N = 46)** | **P-value** |
| --- | --- | --- | --- |
| **Early ECMO initiation (SCAI ≤C)** | 10 (55.6%) | 20 (52.6%) | 0.84 |
| **Frequency of ECMO for CS** |  |  | <0.01 |
| 1–2 cases/year | 4 (22.2%) | 0 (0.0%) |  |
| 3–6. cases/year | 7 (38.9%) | 6 (15.8%) |  |
| 7-12 cases/year | 2 (11.1%) | 8 (21.1%) |  |
| >12 cases/year | 5 (27.8%) | 24 (63.2%) |  |
| **By percent of patients within each center, Median (IQR)**^‡^ | | | |
| **Percent of CS patients treated with ECMO** | 10 (2, 25) | 12.5 (5, 25) | 0.38 |
| **IABP use prior to ECMO** | 10 (5, 25) | 17.5 (5, 50) | 0.25 |
| **pVAD use prior to ECMO** | 20 (10, 50) | 17.5 (10, 25) | 0.46 |
| **LVMU at ECMO initiation for CS** | 25 (15, 50) | 20 (5, 60) | 0.52 |
| **LVMU at any point during VA ECMO for CS** | 50 (10, 75) | 60 (20, 80) | 0.23 |
| **Presence of PAC early in ECMO** | 97.5 (90, 100) | 95 (80, 100) | 0.48 |

† Significance tested using χ²
‡ Significance tested using Wilcoxon–Mann–Whitney
